# Tracking long-distance migration to assess marine pollution impact

**DOI:** 10.1098/rsbl.2011.0880

**Published:** 2011-10-19

**Authors:** William Montevecchi, David Fifield, Chantelle Burke, Stefan Garthe, April Hedd, Jean-François Rail, Gregory Robertson

**Affiliations:** 1 Psychology Department, Memorial University, St. John's, Newfoundland, Canada; 2 Research Technology Centre, University of Kiel, Büsum, Germany; 3 Canadian Wildlife Service, Ste-Foy, Quebec, Canada; 4 Environment Canada, Mount Pearl, Newfoundland, Canada

**Keywords:** tracking, pollution impact, long-distance migrant, seabird mortality, Deepwater Horizon

## Abstract

Animal tracking provides new means to assess far-reaching environmental impacts. In the aftermath of the *Deepwater Horizon* explosion in the Gulf of Mexico, a long-distance migrant, the northern gannet (*Morus bassanus*) suffered the highest oiling among beach-wrecked birds recovered. Analysis of bird-borne tracking data indicated that 25 per cent of their North American population from multiple colonies in eastern Canada migrated to the pollution zone. Findings contrasted sharply with available mark-recapture (band recovery) data. The timing of movement into and out of the Gulf indicates that immature birds would have absorbed most oil-induced mortality. Consequently, one of two outcomes is likely: either a lagged (likely difficult to assess) population decrease, or an undetectable population response buffered by age-related life-history adaptations. Tracking research is especially useful when little information on animal distributions in pollution zones is available, as is the case in the Gulf of Mexico. Ongoing research highlights current risks and conservation concerns.

## 1. Introduction

Advances in animal tracking technology provide new insights into ocean habitat use that can be applied to assess environmental impacts [1]. Long-distance migration and residency patterns improve traditional methods of mark-recapture and mortality assessment and provide benchmarks for recovery efforts [2].

Seabirds are among the most obvious and immediate indicators of wildlife and environmental damage during marine pollution events [3]. In these circumstances, seabird mortality has been assessed by counting dead and dying animals along coasts. These assessments are biased towards animals that die near accessible well-populated coastlines, and as offshore winds and currents can reduce coastal-deposition of carcasses [4] that sink after a few days [5], mortality is inevitably underestimated.

Following the *Deepwater Horizon* explosion on 20 April 2010, bird recoveries in the Gulf of Mexico have been substantial, and all in coastal states (dated 12 May 2011).

For species with more than 100 individuals collected, northern gannets (*Morus bassanus*) rank first in terms of percentage oiled (63%), ahead of royal tern (*Sterna maxima*, 52%), least tern (*Sterna antillarum*, 46%), brown pelicans (*Pelecanus occidentalis*, 41%) and laughing gulls (*Larus atricilla*, 39%; table 1). Of total recoveries, gannets rank third behind resident laughing gulls and brown pelicans.

**Table 1.** Seabird species with more than 100 individuals recovered in the Gulf of Mexico following the Deepwater Horizon explosion on 20 April 2010, as of 12 May 2011. *US Fish and Wildlife Service* (http://www.fws.gov/home/dhoilspill/pdfs/Bird%20Data%20Species%20Spreadsheet%2005122011.pdf dated 12 May 2011).

		total (%)	visibly oiled					unknown oiling			
dead	live		(live that died)	total (%)	not visibly oiled dead	dead	live	(live that died)	total (%)
all species	7258	2121	1062	(541)	2642	3387	873	958	(602)	1229
laughing gull	2981 (41)	1025	355	(198)	1182 (45)	1390	304	371	(266)	409 (33)
brown pelican	826 (11)	152	227	(40)	339 (13)	248	177	149	(87)	239 (19)
no. gannet	475 (7)	225	189	(117)	297 (11)	99	30	107	(58)	79 (6)
royal tern	289 (4)	116	66	(33)	149 (6)	104	19	47	(30)	36 (3)
black skimmer	253 (3)	51	16	(12)	55 (2)	153	40	14	(9)	45 (4)
least tern	106 (1)	46	7	(4)	49 (2)	43	12	3	(1)	14 (1)

The northern gannet, the largest seabird that breeds in the North Atlantic, originates from six eastern Canadian colonies in North America and is the only avian species of solely Canadian origin to have been seriously impacted in the Gulf of Mexico oil spill. Gannets are coastal shelf migrants that winter along the US coast from Maine to Texas. Recent reports based on banding information indicated that only a small portion of the population made long-distance migrations to the Gulf of Mexico [6,7].

To clarify mortality impacts and risks resulting from the Deepwater Horizon blowout, we compare data from bird-borne global location sensors (GLS) and satellite tags (Platform Terminal Transmitters, PTTs) deployed on breeding adult and juvenile gannets from four North American colonies with band recovery data to evaluate preliminary environmental impact.

## 2. Material and methods

### (a) Study sites

**Figure 1.**
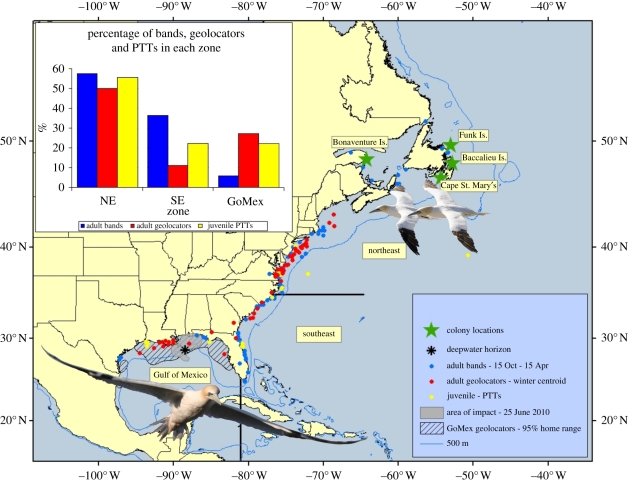
Winter positions of northern gannets from four of six North American colonies where adults and chicks were banded and adults and juveniles were tracked. Mean winter (January–February) positions from adults carrying GLS (2004–2010) and final positions from 18 juveniles with PTTs (2008–2010). Deepwater Horizon site (star) and associated slicks (grey) indicated.

Northern gannets were tracked from colonies at Bonaventure Island (48°29′ N, 64°09′ W), Cape St. Mary's (46°49′ N, 54°49′ W), Baccalieu Island (49°8′ N, 52°47′ W) and Funk Island (49°45′ N, 53°11′ W; figure 1).

### (b) Bird-borne tracking devices and positioning

One hundred and three GLS deployments were made from 2004 to 2010, with 97 Earth and Oceans geo-LT loggers (8.2 g, approx. 0.3% gannet body mass), four British Antarctic Survey MK5 (3.6 g, approx. 0.1% body mass) and two Lotek LTD2400 (8.8 g, approx. 0.3% body mass) devices on plastic leg bands attached to adults with four to five week old chicks. Forty-six winter tracks were obtained. Geo-LTs recorded light level every 30 s, temperature every 120 s; LTD2400 sampled light every 60 s, temperature and pressure every 32 s; no BAS devices were retrieved. From 2008 to 2010, 38 PTTs (32 × KiwiSat 202, 32 g; 6 × AC1, 18 g, approx. 1.2% and 0.7% body mass, respectively) were attached with tape and cable ties to the underside of tails of juvenile gannets; we report on 18 individuals tracked beyond 14 November (median date first juvenile entered the Gulf of Mexico).

Light-based latitude estimates are less accurate than longitude estimates and cannot be determined during solar equinoxes [8], so latitude estimates were obtained or improved by reconciling tag-derived sea surface temperatures (SST) with remotely sensed satellite SST [9]. Positions were smoothed by weighted mean (in a 1 : 2 : 3 : 2 : 1 ratio) of the second previous, previous, current, subsequent and second subsequent position coordinates. All GLS and Advanced Research and Global Observation Satellite (ARGOS)-derived satellite positions were filtered [10]; those requiring unrealistically high travel speed (greater than 84 km h^−1^) from adjacent positions were discarded [11].

### (c) Bands

Banding and recovery records as of 17 January 2006 (13 494 attachments; 832 known-location encounters) were obtained from the North American bird-banding database (http://www.pwrc.usgs.gov/BBL/). Birds were classified as ‘adult’ (*n* = 315) if they were in at least their 5th year when encountered, ‘immature’ (*n* = 469; less than 5 years) or ‘unknown’ (*n* = 48). Encounters were filtered to extract only non-breeding (15 October–15 April; *n* = 288) and winter periods (January–February, *n* = 62). The non-breeding season coincided with typical gannet colony area departure and arrival dates. Winter was restricted to January–February to position GLS birds that had by then ceased large-scale migratory movements.

### (d) Risk and mortality assessments

Tracking and banding data were used to estimate the number of gannets at risk by extrapolation from population estimates ([12,13]; electronic supplementary material, table S1) as a function of the percentage of different age classes using the Gulf of Mexico. The species' winter range was partitioned into three oceanographic zones based on thermal regimes and current systems along the North American shelf—northeast (Gulf of Maine–Chesapeake Bay), southeast (Carolinas–Eastern Florida) and Gulf of Mexico (GoMex; figure 1). We estimate proportions of the population wintering in each sector based on different positioning techniques.

## 3. Results

Year-round band returns suggest that 2.2 per cent of adults and 12.8 per cent of immature birds use the Gulf of Mexico. When analysis is restricted to non-breeding period recoveries, estimates increase to 5.7 per cent of adults and 17.6 per cent of immatures. In contrast, tracking data indicate that much higher percentages of breeding adults (28.3% overall, 13/46 GLS; 25% from Quebec, 7/28 GLS and 27.7% from Newfoundland region 5/18 GLS) and juveniles (22.2%, 4/18 PTTs) wintered in the Gulf of Mexico in at least 1 year (table 2).

**Table 2.** Percentage of North American gannet population using the Gulf of Mexico by age, tracking technique and time of year.

age	North American population	band recoveries (year-round)		band recoveries (15 Oct–15 Apr)		risk estimate (bands)	tracking GLS, PTTs		risk estimate (tracking)
		total (no.)	no. GoMex (%)	total (no.)	no. GoMex (%)		total (no.)	GoMex no. (%)	
AD	233 652^a^	315	7 (2.2%)	7	5 (5.7%)	13 318^d^	46	13 (28.3%)	66 124^f^
immature gannet	236 292^b,c^	469	60 (12.8%)	193	34 (17.6%)	41 587^e^	18	4 (22.2%)	52 509^g^
total	469 950	774	67 (8.7%)	280	39 (13.9%)	54 906	64	17 (26.6%)	118 634

^a^based on ([12,13], electronic supplementary material, table S1).

^b^80% hatching success, 90% fledging success ([14,15], electronic supplementary material, table S1) = 84 115 juveniles.

^c^65% juvenile mortality = 29 441 young in year 1 and 94% survival thereafter through year 6 [14].

^d^233 652 × 0.057 (breeding adults × proportion bands in Gulf of Mexico).

^e^236 292 × 0.176 (immature gannets × proportion bands in Gulf).

^f^233 652 × 0.283 (breeding adults × proportion GLSs in Gulf).

^g^236 292 × 0.222 (immature gannets × proportion PTTs in Gulf).

### (a) Spatial and temporal distributions in the Gulf of Mexico

Gannets concentrated off Louisiana in the Deepwater Horizon pollution area (figure 1). Arrival dates of adults in the area ranged from 11 November to 3 February, exit dates from 23 February to 2 April. A single adult for which we have data in 2010 exited the Gulf on 27 March. Juveniles entered the Gulf from 7 to 28 November, but devices detached before they left the region; immature gannets remain in the Gulf of Mexico much longer than adults ([14]; C. Haney 2011, personal communication).

## 4. Discussion

Northern gannets from four major North American colonies wintered in the zone of Deepwater Horizon pollution which killed many thousands of seabirds including at least many hundreds of gannets and a diversity of other marine wildlife (table 1). Although band recoveries are inherently less reliable than tracking for assessing avian migration patterns and positioning, until now they were the only source of estimates of gannet numbers in the Gulf of Mexico.

Extrapolation from band recoveries indicates that 13 318 adult gannets winter in the Gulf of Mexico, much less than the 66 124 estimate based on GLS data. Gannets defer breeding for 5–7 years [14] and immature birds comprise about half the population. PTT positions suggest an estimated population of 52 509 immature gannets in the Gulf compared with 41 587 extrapolated from banding recoveries. Extrapolating tracking data for all gannet age classes more than doubles the estimated number of birds using the Gulf, from 54 905 to 118 633 birds.

Most bands were applied decades before tracking devices (1960s–1990s versus 2004–2010), so increased occupation of the Gulf of Mexico could reflect a southerly shift of gannets during winter. Such a shift could be related to distributional/abundance changes of an industrially over-exploited key prey, menhanden (*Brevoortia tyrannus*), that is now relatively more abundant in the Gulf of Mexico [16]. In the eastern Atlantic, similar shifts by northern gannets and cape gannets (*Morus capensis*) are associated with pelagic fishing and fish availability [17,18].

Most adult gannets had returned to Canadian colonies by 20 April 2010, although more than 50 000 immature gannets were in the Gulf at the time and suffered oil-related mortality. Hence, two probable outcomes are (i) a lagged (likely difficult to detect) population decrease or (ii) mortality will be buffered by age-related life-history processes [19].

Beyond detection of population-level effects, a body count that underestimates oil-induced mortality carries responsibility for preventable wildlife deaths. In April 2011, at their southernmost colony, Cape St. Mary's Newfoundland, gannets on inaccessible cliff-sites were observed and photographed with dark soiled plumage that looked like oil, but this could not be verified by chemical analysis. Tracking, survival and physiological measurements at gannet colonies during 2011 are evaluating other potential repercussions and informing management about conservation concerns.

## References

[1] Block B. A. 2011 Tracking apex marine predator movements in a dynamic ocean. Nature 475, 86–90 10.1038/nature10082 (doi:10.1038/nature10082) 21697831

[2] Bjorndal K. A. 2011 Better science needed for restoration in the Gulf of Mexico. Science 331, 537–538 10.1126/science.1199935 (doi:10.1126/science.1199935) 21292956

[3] Piatt J. F. Lensink C. J. Butler W. Kendziorek M. Nysewander R. 1990 Immediate impact of the ‘Exxon Valdez’ oil spill on marine birds. Auk 107, 387–397

[4] Ford R. G. Page G. W. Carter H. R. 1987 Estimating mortality of seabirds from oil spills. Am. Petrol. Inst. Oil Spill Conf. Proc. Pub. 4452, 547

[5] Wiese F. K. 2003 Sinking rates of dead birds: improving estimates of seabird mortality due to oiling. Mar. Ornithol. 31, 65–70

[6] Nelson B. 2002 The Atlantic gannet, 2nd edn. Norfolk, VA: Fenix Books

[7] Gaston A. J. Brewer D. Diamond A. W. Woodsworth E. J. Collins B. T. 2008 Canadian Atlas of Bird Banding. Volume 2: Seabirds, 1921–1995. Ottawa, Canada: Canadian Wildlife Service

[8] Hill R. D. 1994 Theory of geolocation by light levels. In Elephant seals: population ecology, behavior, and physiology (eds LeBoeuf B. J. Laws R. M.), pp. 227–236 Berkeley, CA: University California Press

[9] Teo S. L. H. Boustany A. Blackwell S. Walli A. Weng K. C. Block B. A. 2004 Validation of geolcation estimates based on light level and sea surface temperature from electronic tags. Mar. Ecol. Prog. Ser. 283, 81–89 10.3354/meps283081 (doi:10.3354/meps283081)

[10] McConnell B. J. Chambers C. Fedak M. A. 1992 Foraging ecology of southern elephant seals in relation to bathymetry and productivity of the Southern Ocean. Antarct. Sci. 4, 393–398 10.1017/S0954102092000580 (doi:10.1017/S0954102092000580)

[11] Garthe S. Montevecchi W. A. Davoren G. K. 2007 Flight destinations and foraging behavior of gannets preying on small forage fishes. Deep-Sea Res. 54, 311–320 10.1016/j.dsr2.2006.11.008 (doi:10.1016/j.dsr2.2006.11.008)

[12] Chardine J. W. 2000 Census of northern gannet colonies in the Atlantic Region, 1999. CWS Tech. Rep. 361, 1–16

[13] Chapdelaine G. Brousseau P. Rail J. F. 2010 Banque Informatisée des Oiseaux Marins du Québec (BIOMQ) (Environment Canada, Quebec, CWS).

[14] Mowbray T. B. 2002 Northern Gannet, no. 693 (The Birds of North America) (http://bna.birds.cornell.edu).

[15] Montevecchi W. A. Wells J. 1984 Fledging success of gannets from different nest-sites. Bird Behav. 5, 90–95

[16] Franklin H. B. 2007 The most important fish in the sea. Washington, DC: Island Press

[17] Kubetzki U. Garthe S. Fifield D. A. Mendel B. Furness R. W. 2009 Individual migratory schedules and wintering areas of gannets. Mar. Ecol. Prog. Ser. 391, 257–265 10.3354/meps08254 (doi:10.3354/meps08254)

[18] Crawford R. J. M. Dundee B. L. Dyer B. M. Klages N. T. Meÿer M. A. Upfold L. 2007 Trends in numbers of Cape Gannets, 1956/57–2006/06, with consideration of food and other factors. ICES J. Mar. Sci. 64, 169–177 10.1093/icesjms/fsm009 (doi:10.1093/icesjms/fsm009)

[19] Votier S. Birkhead T. R. Oro D. Trinder M. Grantham M. J. McCleery R. H. Hatchwell B. J. 2008 Recruitment and survival of immature seabirds in relation to oil spills and climate variability. J. Anim. Ecol. 77, 974–983 10.1111/j.1365-2656.2008.01421.x (doi:10.1111/j.1365-2656.2008.01421.x) 18624739

